# The complete mitochondrial genome of the meerkat (*Suricata suricatta*) and its phylogenetic relationship with other feliform species

**DOI:** 10.1080/23802359.2020.1726221

**Published:** 2020-02-11

**Authors:** Lorena Derežanin, Jörns Fickel, Daniel Förster

**Affiliations:** Evolutionary Genetics Department, Leibniz Institute for Zoo and Wildlife Research (IZW), Berlin, Germany

**Keywords:** Mitogenome, Herpestidae, *Suricata suricatta*, phylogenetics, Feliformia

## Abstract

The meerkat, *Suricata suricatta*, is a highly social member of the mongoose family (Herpestidae) and the only extant species of the genus *Suricata*. We present the first complete mitochondrial genome of the meerkat, assembled with a seed-and-extend algorithm using three closely related species as references. Phylogenetic analyses using 22 mitochondrial genome sequences confirm the position of meerkat within the Herpestidae family and the Feliformia, a suborder of Carnivora, with high support values. This position is in good agreement with formerly conducted studies based on a small number of mitochondrial and nuclear gene fragments. Our complete mitochondrial genome represents a valuable resource for further phylogenetic studies, especially of the underrepresented members of the Herpestidae family.

The meerkat (*Suricata suricatta*) is a small carnivoran species occurring in southwestern Angola, Namibia, Botswana, and South Africa (Macdonald 2013). It is a member of the mongoose family (Herpestidae) and the only extant species of the genus *Suricata.*

Meerkats are widespread in arid habitats such as shrubland but are absent from true deserts and mountainous areas (Macdonald 2013). They feed mostly on invertebrates and are highly social. Population densities are influenced by rainfall and predation and fluctuate considerably across meerkats’ range (Clutton-Brock et al. 1999). Still, current populations appear stable and the IUCN assessment for the species is ‘least concern’.

A tissue sample (muscle) was obtained from a captive male from Poznan Zoo (52°24′03.2″N, 16°59′50.3″E), collected during necropsy conducted at the Leibniz Institute for Zoo and Wildlife Research, Berlin, Germany, where it is permanently stored in the IZW DNA Archive with the sample ID: 303_18. We extracted genomic DNA using the QIAGEN Blood & Cell Culture DNA kit following the manufacturer’s instructions. An Illumina TrueSeq DNA PCR-free library was built with an average insert size of 550 bp and was sequenced (PE 150) on the Illumina HiSeq.

We performed adapter clipping and quality trimming of raw reads with TrimGalore v.0.6.4 (https://github.com/FelixKrueger/TrimGalore). The complete circular mitogenome was assembled using NOVOPlasty v.3.7 (Dierckxsens, Mardulyn and Smits, 2017). Three independent runs were performed, each employing a different mitochondrial reference sequence: *Herpestes javanicus* (GenBank: KY117548.1), *Herpestes brachyurus hosei* (GenBank: KY117547.1), and *Mungotictis decemlineata* (GenBank: NC_027828.1). The meerkat assemblies generated using the three different references were identical.

We mapped 85,754 properly paired reads to mitogenome with Bowtie2 v.2.3.5.1 (Langmead and Salzberg 2012) and called consensus sequence with a minimum read coverage of 30× and a 100% base call threshold in Geneious v.9.0.5 (Kearse et al. 2012). The resultant circular sequence, 16,914 bp in length (GenBank Accession number: MN854374), was annotated using MITOS (Bernt et al. 2013). The complete mitogenome consists of a control region and a conserved set of 37 genes including 13 protein-coding genes with their expected open reading frames, 22 tRNA and 2 rRNA genes typical of vertebrate mitochondrial genomes. Lengths of protein-coding genes in the meerkat mitogenome were the same as in published mitogenomes of other herpestid species.

We performed multiple sequence alignment with 21 representatives of the order Carnivora using MAFFT v.7.407 (Katoh and Standley, 2013), with gray wolf (*C. lupus*) used as an outgroup. The control region (1508 bp) was excluded from further analysis. The maximum-likelihood tree was constructed using RAxML v.8.2.9 (Stamatakis 2014) applying GTR + G+I as the substitution model, as determined using PartitionFinder v.2.1.1 (Lanfear et al. 2012, 2017).

Our phylogenetic analysis supports the placement of the meerkat within the Herpestidae family, forming a sister clade to genus Herpestes with high confidence (100%) based on 1000 bootstrap replicates (Figure 1). This is in concordance with previously published analyses derived from a limited number of mitochondrial and nuclear genes (Patou et al. 2009).

**Figure 1.**
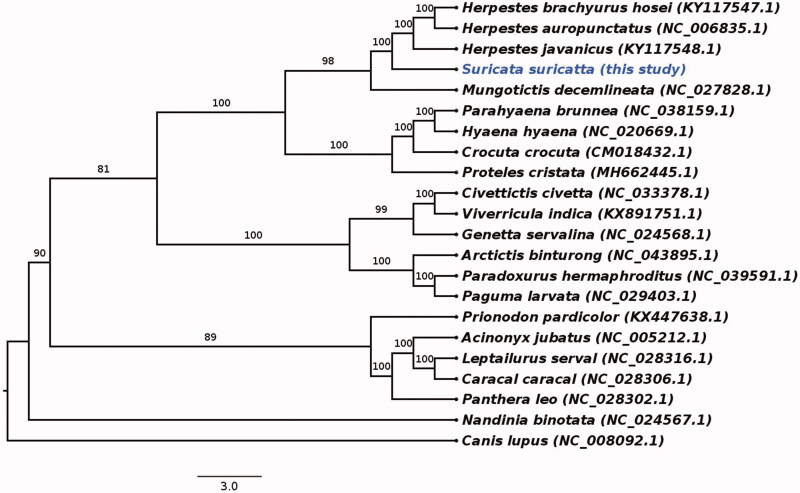
The phylogenetic relationship of *Suricata suricatta* with genus *Herpestes* and other clades within Feliformia inferred from maximum-likelihood analysis based on mitogenome sequences (alignment length = 15,406 bp). Numbers on branches represent bootstrap values. NCBI GenBank accession numbers are given in parentheses.

## References

[CIT0001] Bernt M, Donath A, Jühling F, Externbrink F, Florentz C, Fritzsch G, Pütz J, Middendorf M, Stadler PF. 2013. MITOS: improved de novo metazoan mitochondrial genome annotation. Mol Phylogenet Evol. 69(2):313–319. 22982435 10.1016/j.ympev.2012.08.023

[CIT0002] Clutton-Brock TH, Gaynor D, McIlrath GM, Maccoll ADC, Kansky R, Chadwick P, Manser M, Skinner JD, Brotherton PNM. 1999. Predation, group size and mortality in a cooperative mongoose, *Suricata suricatta*. J Anim Ecology. 68(4):672–683.

[CIT0003] Dierckxsens N, Mardulyn P, Smits G. 2017. NOVOPlasty: de novo assembly of organelle genomes from whole genome data. Nucleic Acids Res. 45(4):e18. 28204566 10.1093/nar/gkw955 PMC5389512

[CIT0004] Katoh K, Standley DM. 2013. MAFFT multiple sequence alignment software version 7: improvements in performance and usability. Mol Biol Evol. 30(4):772–780. 23329690 10.1093/molbev/mst010 PMC3603318

[CIT0005] Kearse M, Moir R, Wilson A, Stones-Havas S, Cheung M, Sturrock S, Buxton S, Cooper A, Markowitz S, Duran C, et al. 2012. Geneious Basic: an integrated and extendable desktop software platform for the organization and analysis of sequence data. Bioinformatics. 28(12):1647–1649. 22543367 10.1093/bioinformatics/bts199 PMC3371832

[CIT0006] Lanfear R, Calcott B, Ho SYW, Guindon S. 2012. Partitionfinder: combined selection of partitioning schemes and substitution models for phylogenetic analyses. Mol Biol Evol. 29(6):1695–1701. 22319168 10.1093/molbev/mss020

[CIT0007] Lanfear R, Frandsen P B, Wright A M, Senfeld T, Calcott B. 2017. PartitionFinder 2: new methods for selecting partitioned models of evolution for molecular and morphological phylogenetic analyses. Mol Biol Evol. 34(3):772–773. 28013191 10.1093/molbev/msw260

[CIT0008] Langmead B, Salzberg S. L. 2012. Fast gapped-read alignment with Bowtie 2. Nat Methods. 9(4):357–359. 22388286 10.1038/nmeth.1923 PMC3322381

[CIT0009] Macdonald D. W. 2013. *Suricata suricatta* Meerkat (Suricate). In: Kingdon, J. and Hoffmann, M., editors, The Mammals of Africa, volume V: carnivores, pangolins, equids and rhinoceroses. London, UK: Bloomsbury Publishing; p. 347–352.

[CIT0010] Patou M-L, Mclenachan PA, Morley CG, Couloux A, Jennings AP, Veron G. 2009. Molecular phylogeny of the Herpestidae (Mammalia, Carnivora) with a special emphasis on the Asian Herpestes. Mol Phylogenet Evol. 53(1):69–80. 19520178 10.1016/j.ympev.2009.05.038

[CIT0011] Stamatakis A. 2014. RAxML version 8: a tool for phylogenetic analysis and post-analysis of large phylogenies. Bioinformatics. 30(9):1312–1313. 24451623 10.1093/bioinformatics/btu033 PMC3998144

